# Effects of Scan Speed on Crack Elimination, Microstructural Evolution, and Mechanical Properties of IN738LC Alloy Processed by Laser Powder Bed Fusion

**DOI:** 10.3390/ma19091727

**Published:** 2026-04-24

**Authors:** Pengju Wang, Jingguang Du, Linqing Liu, Yang Wei, Wenqing Yang, Yang Li, Changjun Han, Xusheng Yang, Hua Tan, Leilei Wang, Yongqiang Yang, Di Wang

**Affiliations:** 1 School of Mechanical and Automotive Engineering, South China University of Technology, Guangzhou 510641, China; 2 Department of Industrial and Systems Engineering, The Hong Kong Polytechnic University, Hong Kong 999077, China; wenqing.yang@connect.polyu.hk (W.Y.); xusheng.yang@polyu.edu.hk (X.Y.); 3 State Key Laboratory of Solidification Processing, Northwestern Polytechnical University, Xi’an 710072, China; 4 College of Materials Science and Technology, Nanjing University of Aeronautics and Astronautics, Nanjing 211106, China

**Keywords:** laser powder bed fusion, IN738LC alloy, crack elimination, microstructural evolution, mechanical properties

## Abstract

Cracking represents a critical issue in γ’-strengthened Ni-based superalloys processed via laser powder bed fusion. This study systematically investigated the influence of scan speed (800–1200 mm/s) on the crack elimination mechanism, microstructural evolution, and mechanical properties of LPBF-processed IN738LC alloy. Near-defect-free IN738LC parts were successfully produced with a relative density of 99.6% and a crack density of only 0.025%. The results indicate that as the scan speed increased from 800 mm/s to 1100 mm/s, a flatter melt pool (S4) was obtained, which reduced the proportion of high-angle grain boundaries. The cooling rate also increased from 13.68 K/μs to 15.96 K/μs, promoting grain refinement and the dispersion precipitation of MC carbides. The refined grains effectively suppressed stress concentration and inhibited crack propagation along grain boundaries. The optimized process (1100 mm/s) achieved optimal comprehensive mechanical properties. Compared to a scan speed of 800 mm/s, the ultimate tensile strength, yield strength, and elongation at room-temperature increased from 1075 MPa, 820 MPa, and 13.2% to 1179 MPa, 871 MPa, and 21.1%, respectively, while hardness increased from 365 HV_1.0_ to 387 HV_1.0_. This study demonstrated that the microstructure and mechanical properties of LPBF-processed IN738LC alloy can be tailored via controlling the thermal history of the melt pool, providing a foundation for processing high-crack-sensitivity alloys utilizing laser powder bed fusion.

## 1. Introduction

IN738LC alloy represents a typical γ’-phase precipitation-hardened Ni-based superalloy that maintains excellent high-temperature strength and creep resistance at 850 °C and even higher temperatures. Consequently, it is extensively utilized in key components such as turbine blades, guide vanes, and industrial gas turbines [1,2]. Such components typically feature intricate internal cooling structures and demand extremely high geometric precision, making it challenging for traditional manufacturing methods like casting and forging to simultaneously achieve complex structural formation and the desired material properties [3]. Laser powder bed fusion (LPBF) represents an advanced additive manufacturing technology based on the principle of “layered manufacturing and sequential deposition.” It enables the direct fabrication of intricate metal parts with high density and superior mechanical properties [4], offering a novel technological approach for producing high-performance Ni-based superalloy components [5,6].

However, unlike Ni-based superalloys such as IN718 and IN625, which have successfully achieved printable LPBF fabrication, IN738LC is highly susceptible to crack initiation during rapid solidification, severely limiting its development and application in LPBF. The high Al and Ti (Ti + Al > 6 wt%) content significantly broadens the solidification temperature range of IN738LC, prolongs the duration of the mush zone, and promotes the formation of low-melting-point liquid films at grain boundaries [7,8]. In the LPBF process, the liquid film is sheared under thermal stress induced by a high temperature gradient and rapid cooling; combined with insufficient inter-dendritic filling, this ultimately leads to crack formation [9].

Regarding the formation of cracks in the LPBF-processed IN738LC alloy, extensive research was conducted by scholars worldwide, focusing on crack suppression methods such as alloy composition adjustment [10,11], process parameter optimization [7], scan strategy optimization [12,13], and substrate preheating [14,15]. Zhang et al. [16] employed the Scheil–Gulliver solidification model to analyze the influence of Mn and Si elements on crack sensitivity. It was found that reducing the Mn + Si content could minimize the formation of low-melting-point phases at grain boundaries and reduce the solidification temperature range, thereby suppressing hot cracking. Guo et al. [17] achieved in situ formation of Y_4_Al_2_O_9_ particles by adding Y_2_O_3_ nanoparticles, effectively suppressing Zr segregation at grain boundaries and eliminating cracks. Cloots et al. [12] utilized APT technology to reveal that Zr segregation at grain boundaries leads to the formation of low-melting-point liquid films at grain boundaries. The direction of dendrite growth in the shallow melt pool obtained by increasing the scan speed was consistent, reducing the proportion of high-angle grain boundaries, thereby inhibiting cracking. Guo et al. [18] pointed out that at low energy input, unfused pores induce stress concentration that led to crack initiation, while at high energy input, cracks propagate along high-angle grain boundaries. Al-rich oxides and silicide particles rich in Ta, W, and Si are present near cracks. These brittle phases disrupt the continuity of the liquid film during late solidification, thereby increasing the risk of cracking. Xu et al. [19] systematically examined the impact of interlayer rotation angle on crack suppression, finding that a 67° rotation strategy achieved near-crack-free forming through grain refinement and residual stress reduction. Lam et al. [20] compared multiple scan strategies and found that the 90° rotation strategy reduced the proportion of high-angle grain boundaries (HAGBs) compared to the conventional 67° rotation, thereby decreasing crack density. Additionally, substrate preheating was confirmed as an effective means of crack suppression. Chen et al. [8] demonstrated that, compared to 200 °C, preheating at 700 °C significantly reduced residual stress and the proportion of HAGBs, thereby suppressing thermal crack formation, but current commercial LPBF equipment has difficulty achieving this temperature, which limits the widespread application of this method.

It is notable that as one of the core parameters in LPBF, scan speed directly influences the thermal behavior of the melt pool, microstructure and corresponding mechanical properties [21]. Studies indicate that scan speed significantly affects the temperature gradient and cooling rate in the melt pool, thereby impacting grain morphology and grain size [15]. However, research on controlling melt pool thermal behavior through scan speed to influence the dendritic growth and grain morphology of the IN738LC alloy, and ultimately its cracking behavior, remains relatively limited.

In this study, the impact of scan speed upon crack elimination, microstructural evolution, and the mechanical properties of IN738LC alloy was systematically explored. By optimizing the scan speed, the printability of the IN738LC alloy was significantly improved, achieving a favorable balance of tensile strength and ductility. A three-dimensional transient numerical model was developed to analyze the effect of scan speed on the thermal history of the melt pool. By analyzing dendrite epitaxial growth and grain morphology within the melt pool, the mechanisms by which scan speed affects cracking behavior and mechanical properties were revealed. This research provides the theoretical basis and process guidance for LPBF-processed high-crack-sensitivity Ni-based superalloys.

## 2. Experiment and Simulation

### 2.1. Powder Characterization

**Figure 1:**
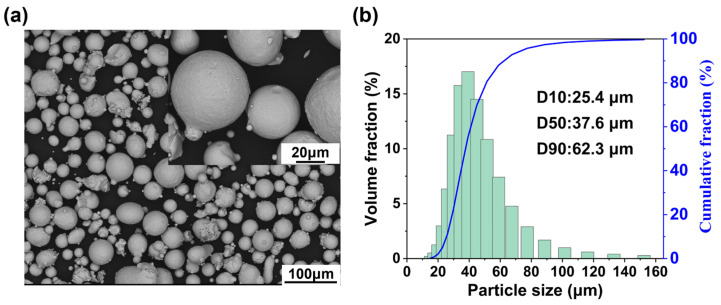
(**a**) Powder morphology; (**b**) particle size distribution of the powder.

IN738LC powder (GRAM Technology Ltd., Beijing, China) for LPBF was produced through gas atomization, and its composition is shown in Table 1 (chemical composition data provided by the powder supply company). Scanning electron microscopy (SEM) revealed that the powder particles are nearly spherical, with tiny satellite particles adhering to the surfaces of large particles (Figure 1a). Laser particle analyzer (LPA) measurements indicate the average particle diameter of 41.9 μm and a D50 value of 37.6 μm (Figure 1b).

### 2.2. LPBF Process

**Figure 2:**
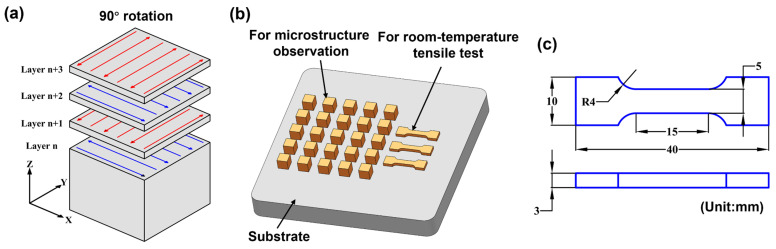
(**a**) Schematic of laser scan strategy; (**b**) schematic of microstructure characterization and room-temperature tensile specimen; (**c**) detailed dimension diagram of tensile test specimen.

The LPBF forming equipment (DiMetal-280, South China University of Technology, Guangzhou, China) is fitted with a 500 W IPG-YLR-500 continuous fiber laser, featuring the beam diameter of 90 μm at the focal plane. To prevent sample oxidation, the entire LPBF process is operated in an argon atmosphere (pressure below 0.08 bar), and an oxygen analyzer is used to maintain the oxygen content within the chamber below 100 ppm. To ensure excellent material bonding, the samples are fabricated on 316L stainless steel substrates at room-temperature. Figure 2a illustrates the orthogonal scan strategy implemented in this study, employing a reciprocating scan pattern with a 90° rotation between adjacent layers. To optimize process parameters, this study was conducted within the ranges of laser power (*P* = 175–275 W) and scan speed (*v* = 800–1200 mm/s), while other parameters—including hatch distance (*h* = 100 μm) and layer thickness (*t* = 30 μm)—remained constant. The volume energy density (*VED*) is the combination of laser power, scan speed, hatch spacing, and layer thickness, which can be expressed as [22]

(1) $$VED=\frac{P}{vht}$$

As shown in Table 2, the range of calculated *VED* values is 48.6 to 114.6 J/mm^3^. To investigate the effect of scan speed on LPBF-processed IN738LC alloy, five specimens prepared under 225 W conditions (The sample exhibits the best overall forming quality under this laser power) were designated as S1, S2, S3, S4, and S5 and subjected to subsequent characterization and mechanical property testing. Specimens measuring 10 × 10 × 10 mm^3^ and 40 × 10 × 3 mm^3^ were fabricated. The prepared specimens were separated from the substrate using wire cutting and were used for relative-density measurements, microstructure observation, and room-temperature tensile testing, as shown in Figure 2b. Figure 2c displays the detailed dimensions of the tensile test specimens.

### 2.3. Crack Characterization

**Figure 3:**
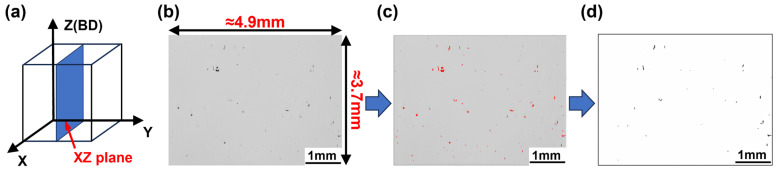
(**a**) Schematic of crack measurement of specimen; (**b**) OM observation of defects; (**c**) defect identification in ImageJ 1.54f software; (**d**) crack identification diagram after filtering of pores.

Cracks were analyzed using an optical microscope (OM, DMI5000 M, Leica, Wetzlar, Germany) along with ImageJ 1.54f software. As shown in Figure 3a, four characteristic regions were selected in the XZ plane of the sample. The observation position should be 0.5 mm from the edge of the sample to prevent cracks at the edge from impacting the result. Images were acquired in bright-field mode at 50× magnification, with the light intensity fixed to avoid artificial variations in contrast. The acquired OM images covered an area of approximately 4.9 mm × 3.7 mm (Figure 3b). All defects were extracted from the images using ImageJ 1.54f software with appropriate threshold settings (Figure 3c). Following the methodology of Liu et al. [22], cracks were screened based on the circularity coefficient (C), defined as

(2) $$C=4\pi (\frac{A}{p^{2}})$$

where the *A* is the defect area and *p* is the defect perimeter. Cracks are identified under the constraint *C* < 0.5, then they are converted into line segments, and their lengths are calculated (Figure 3d). Crack density was defined by the overall length of crack within a unit area (unit: mm/mm^2^) [23], and the crack density for each sample was calculated as the average of measurements from four areas.

### 2.4. Microstructural Characterization and Mechanical Testing

The relative density of samples after surface grinding was computed utilizing the Archimedes principle. Distilled water was used as the immersion medium, and the temperature was maintained at 25 °C. Each sample was measured three times to guarantee data reliability, and the average was calculated. Samples were progressively polished using 180–3000 grit sandpaper, then polished with 0.5 μm aluminum oxide polishing powder and 0.06 μm colloidal silica suspension until a mirror finish was achieved. The polished samples were etched for 1 min at 25 °C in an etching solution (H_2_O: HCl: HNO_3_ = 3:3:1). The microstructure of the sample was characterized via OM and a field emission SEM (FEI Nova nano 430, FEI, Eindhoven, The Netherlands) fitted with an energy-dispersive X-ray spectroscopy (EDS) system. Phase composition was analyzed via X-ray diffraction (XRD, X’pert Powder, PANalytical, Almelo, The Netherlands) over a scan scope of 2θ = 30–120°, with a step size of 0.013° and a scan speed of 12°/min. Grain morphology was measured via electron backscatter diffraction (EBSD) utilizing a field emission SEM. The data were collected at 15 kV acceleration voltage with a 0.5 μm step size and were analyzed using Oxford Instruments AztecCrystal 2.1 software. Tensile tests were conducted using the CMT5105 testing machine (Zhuhai SUST Co., Ltd., Zhuhai, China) equipped with an extensometer, with a tensile rate of 0.033 min^−1^; the tensile direction is perpendicular to the build direction (BD). Each test condition was conducted three times, and the mean value and standard deviation were calculated. The fracture morphology of the tensile specimens was analyzed using SEM to reveal the fracture morphology. Hardness measurements were performed utilizing a HVS-10 Vickers hardness tester (SMTMF, Shanghai, China), with a load of 1 kgf applied and a holding time of 15 s. Each sample underwent ten measurements, and the average value and standard deviation were calculated.

### 2.5. Finite Element Analysis

(1) Thermal analysis

In the LPBF process, the metal powder is quickly heated and melted by the laser beam as the laser moves. After the laser moves away, it rapidly cools and solidifies into a solid entity. This process constitutes a nonlinear transient heat transfer phenomenon, governed by the classical Fourier heat transfer equation [24,25]:

(3) $$\rho c\frac{\partial T}{\partial t}=\frac{\partial }{\partial x}(k\frac{\partial T}{\partial x})+\frac{\partial }{\partial y}(k\frac{\partial T}{\partial y})+\frac{\partial }{\partial z}(k\frac{\partial T}{\partial z})+q$$

where *ρ* and *c* represent the density and specific heat capacity of the material, respectively; *k* and *T* represent the thermal conductivity and the instantaneous temperature of the material, respectively; and *q* represents the heat flux. To solve the thermal conduction equation, the initial and boundary conditions for the LPBF process must be specified. Assuming the initial temperature of the powder is *T*_0_, the initial condition is

(4) $$T{\left(x,y,z\right)}_{t=0}=T_{0}$$

**Figure 4:**
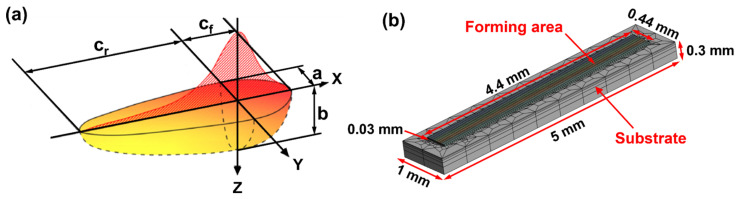
(**a**) Schematic of a double-ellipsoidal heat source; (**b**) the finite element model.

Under the experimental conditions, *T*_0_ = 293 K (20 °C). In the LPBF process, the metal powder surface absorbs laser irradiation energy. Additionally, thermal conduction, convection, and radiation occur within the powder bed. The boundary conditions can be described as [26]

(5) $$k\frac{\partial T}{\partial z}+h(T-T_{E})+\sigma \varepsilon (T^{4}{-T_{E}}^{4})-q=0$$

where *z* and *h* represent the normal direction of the heat flux plane and the convective heat transfer coefficient, respectively; *T_E_* and *σ* are the ambient temperature and the Stefan–Boltzmann constant (approximately 5.67 × 10^−8^ W/(m^2^·K^4^) [24], and *ε* represents the thermal radiation coefficient of the material. To predict transient temperature and heat flow distribution at nodes during the LPBF process, a double-ellipsoidal heat source was selected as the thermal input model. Developed as an enhancement of the Gaussian heat source, this model has been widely applied in welding and additive manufacturing [27]. Its geometric representation is shown in Figure 4a, and its distribution of energy can be expressed by the following equation:

(6) $$Q_{f}(x,y,z)=\frac{6\sqrt{3}f_{f}P}{abc\pi \sqrt{\pi }}e^{\left(-\frac{3x^{2}}{{c_{f}}^{2}}\right)}e^{\left(-\frac{3y^{2}}{a^{2}}\right)}e^{\left(-\frac{3z^{2}}{b^{2}}\right)}(x\geq 0)$$

(7) $$Q_{r}(x,y,z)=\frac{6\sqrt{3}f_{r}P}{abc\pi \sqrt{\pi }}e^{\left(-\frac{3x^{2}}{{c_{r}}^{2}}\right)}e^{\left(-\frac{3y^{2}}{a^{2}}\right)}e^{\left(-\frac{3z^{2}}{b^{2}}\right)}(x<0)$$

where *Q* represents power density, *P* denotes laser power, and *f_f/r_* represents the energy ratio between the front and rear ellipsoidal halves, with their sum equaling 2. *A* represents the laser absorption coefficient of the metal powder, *a* is the width of the heat flow, and *b* is the depth of the heat flow. In this work, the parameters of the double-ellipsoidal heat source are *f_f_* = 0.67, *f_r_* = 1.33, *c_f_* = 0.05 mm, *c_r_* = 0.1 mm, *a* = *b* = 0.07 mm [13].

(2) Finite element model and material physical properties

**Figure 5:**
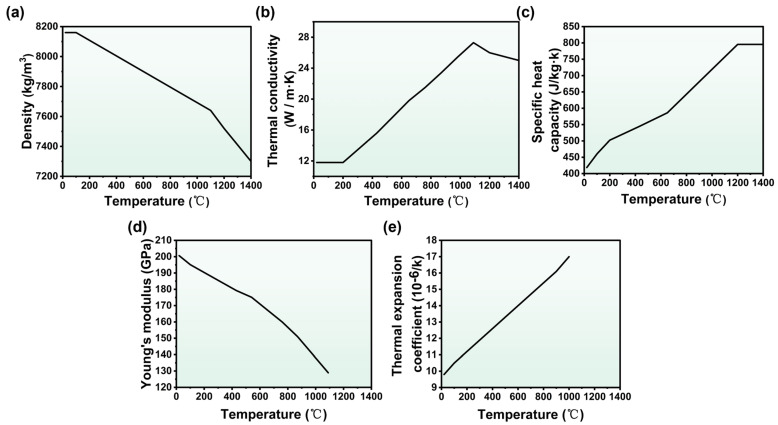
Physical properties of IN738LC alloy varying with temperature: (**a**) Density; (**b**) thermal conductivity; (**c**) specific heat capacity; (**d**) Young’s modulus; (**e**) thermal expansion coefficient.

A three-dimensional finite element model was developed using commercial software ANSYS 2025R2 to explore the effect of scan speed upon the thermal history of the melt pool. In the LPBF process, the laser beam interaction time is exceedingly brief, producing a minimal heat-affected zone. Beyond a certain model size, the mutual influence between different regions diminishes significantly [25]. To reduce computational costs, a one-layer, four-melt-channel finite element model was established. The forming area dimensions were set to 4.4 × 0.44 × 0.03 mm^3^, with the substrate size at 5 × 1 × 0.3 mm^3^, as shown in Figure 4b. The forming area was divided into hexagonal meshes of 0.04 × 0.025 × 0.015 mm^3^, while the substrate was divided into tetrahedral meshes. The model comprised 32,190 meshes and 40,269 points. The majority of physical properties of the IN738LC alloy utilized in this research are temperature-dependent [28,29,30], including density, thermal conductivity, specific heat capacity, Young’s modulus, and thermal expansion coefficient (Figure 5). Additionally, other temperature-independent physical properties are listed in Table 3.

## 3. Results

### 3.1. Densification Behavior and Defect Analysis

**Figure 6:**
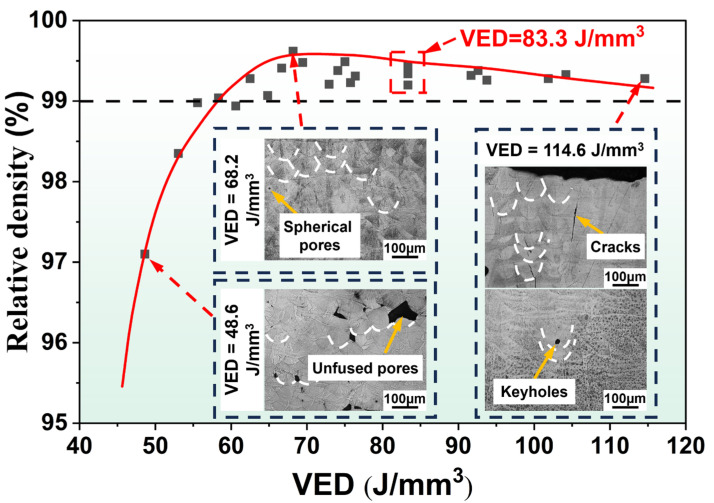
Relative density of LPBF-processed IN738LC samples with different *VEDs*.

Figure 6 shows the relative density of samples with varying *VEDs*. When *VED* was low (48.6 J/mm^3^), insufficient laser energy input led to numerous unfused pores [32], resulting in low relative density (<99%). As *VED* increased (68.2 J/mm^3^), unfused pores gradually disappeared, leaving only a few spherical pores, resulting in increased relative density (>99%). When *VED* further increased (>93.8 J/mm^3^), keyhole and crack defects begin forming within the specimen [15,33], causing relative density to decrease. Nevertheless, IN738LC achieved high-density forming within a broad process window, reaching a maximum relative density of 99.6% at *VED* = 68.2 J/mm^3^ (S4). Furthermore, at *VED* = 83.3 J/mm^3^, samples with the same *VED* exhibited differing relative density. This discrepancy stemmed from the differential impact of laser power and scan speed upon relative density [8,15]. Consequently, the characteristics of defects under varying scan speeds were further investigated.

**Figure 7:**
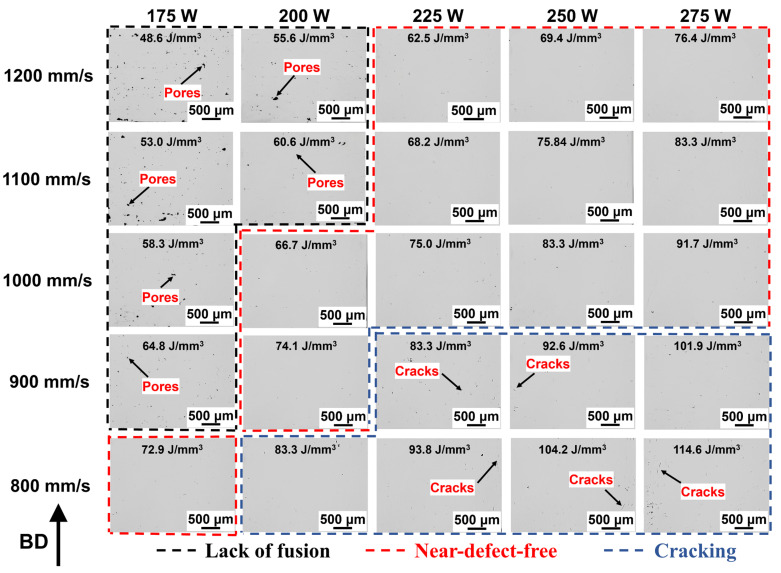
OM images of IN738LC samples with different process parameters.

Figure 7 displays micrographs of IN738LC samples fabricated with a laser power of 175–275 W and a scan speed of 800–1400 mm/s. The micrographs were divided into three regions: lack-of-fusion area, near-defect-free area, and cracking area. When *VED* = 48.6–64.8 J/mm^3^, the lack-of-fusion area exhibited numerous unfused pores, resulting in a relative density of only 97.1–99.1%. When *VED* = 62.5–91.7 J/mm^3^, the near-defect-free area exhibited only minor fine cracks and pores, with relative density increasing to 99.2–99.6%. As *VED* increased to 83.3–114.6 J/mm^3^, the cracking region exhibited more cracks accompanied by minor keyhole defects, resulting in a decrease in relative density to 99.3–99.4% (Figure 6).

**Figure 8:**
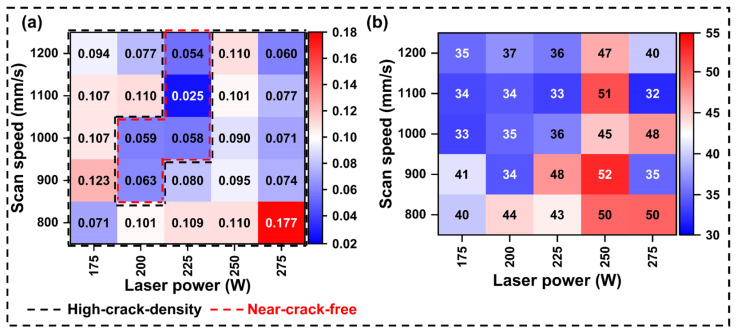
Statistical data of (**a**) crack density and (**b**) average crack length for IN738LC samples at different process parameters.

As a commonly utilized defect characterization method, crack density analysis was extensively employed for crack-sensitive Ni-based superalloys such as IN738LC [8,12,20,23]. Figure 8a demonstrates the statistical results of crack density for samples processed with different parameters. Notably, the crack density distribution can be divided into a high-crack-density region and a near-crack-free region. The crack density in the high-crack-density region ranged from 0.060 to 0.177 mm/mm^2^. Conversely, near-crack-free forming was achieved in the near-crack-free region, with a minimum crack density of only 0.025 mm/mm^2^ (S4). Figure 8b displays the average crack lengths of IN738LC specimens under different process parameters. Notably, specimens with a high *VED* (high laser power combined with low scan speed) typically exhibit higher average crack lengths. Conversely, specimens with a low VED (low laser power and high scanning speed) show lower average crack lengths. S4 demonstrated an average crack length of only 33 μm.

In summary, although pore and crack defects both exhibited high sensitivity to process parameters, LPBF-processed IN738LC alloy still demonstrated favorable printability within a broad process window (62.5–75.0 J/mm^3^). Among the samples, the set processed at a laser power of 225 W achieved the most ideal forming results. Based on this, subsequent studies focused on investigating the effect of scan speed on the microstructure of the samples (S1–S5). Notably, when the scan speed was 1100 mm/s, S4 exhibited the highest relative density and the lowest crack density. Therefore, S1 was used as the control group, and the characterization focused primarily on S1 and S4.

### 3.2. Melt Pool Morphology and Microstructure

(1) Melt pool size and morphology

**Figure 9:**
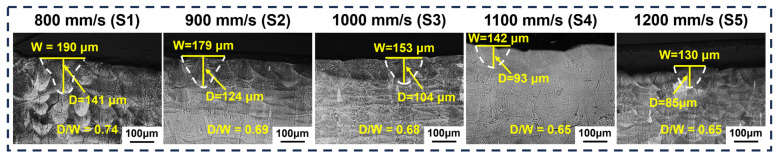
Images of melt pool size and morphology for samples with different scan speeds.

The size and morphology of the melt pool at different scan speeds are shown in Figure 9. It was observed that with the scan speed increased from 800 mm/s to 1200 mm/s, the reduced laser-powder interaction time led to reduced energy input, causing melt pool depth and width to decrease from 141 μm and 190 μm to 85 μm and 130 μm, respectively. Furthermore, as another characteristic of the melt pool, its morphology was primarily influenced by the depth-to-width ratio (D/W) [34,35]. The melt pool morphology also exhibited significant changes with increasing scan speed. With a scan speed of 800 mm/s, the D/W of the melt pool is 0.74, and the melt pool morphology was “wine cup-shaped” (S1). As the scan speed increased to 1000 mm/s, the D/W of the melt pool was 0.68, and the morphology transformed into being “inverted pear-shaped” (S3). Further increasing the scan speed to 1100 mm/s resulted in a D/W of 0.65, where the morphology changed to “semicircle-shaped” (S4 and S5) [16].

(2) Phase composition

**Figure 10:**
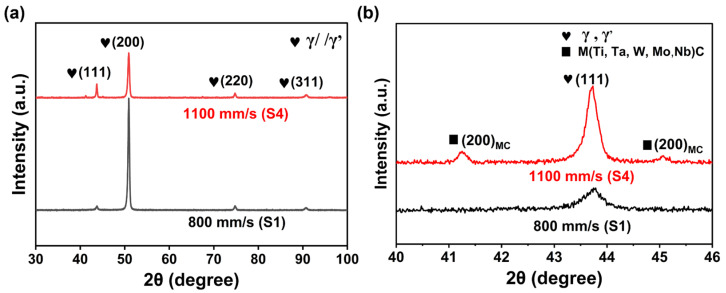
(**a**) XRD spectrum of the LPBF-processed IN738LC sample; (**b**) detail magnification of the X-ray diffraction pattern.

Figure 10 displays the XRD spectrum of the LPBF-processed IN738LC alloy. Due to the highly similar lattice constants of the γ′ and γ phases, their diffraction peaks exhibited near-complete overlap, making it impossible to distinguish their individual diffraction characteristics [36]. The diffraction pattern results indicated that strong (200) diffraction peaks and weak (111), (220), and (311) diffraction peaks of the γ/γ’ phase were detected under both scan speed conditions. This phenomenon was consistent with the findings of previous studies [10,36]. Notably, unlike the strong (200) diffraction peak observed in S1, the (200) peak in the S4 was weakened, while the (111), (220), and (311) peaks were enhanced (Figure 10a). This variation could be attributed to changes in the thermal history of the melt pool at different scan speeds [9,21,36]. In addition, S1 detected (111) diffraction peaks (Figure 10b), which previous studies have identified as characteristic of MC carbides [10,36,37,38]. However, the peaks of MC carbides were not found in the pattern of S1.

(3) Grain morphology and precipitated phase

**Figure 11:**
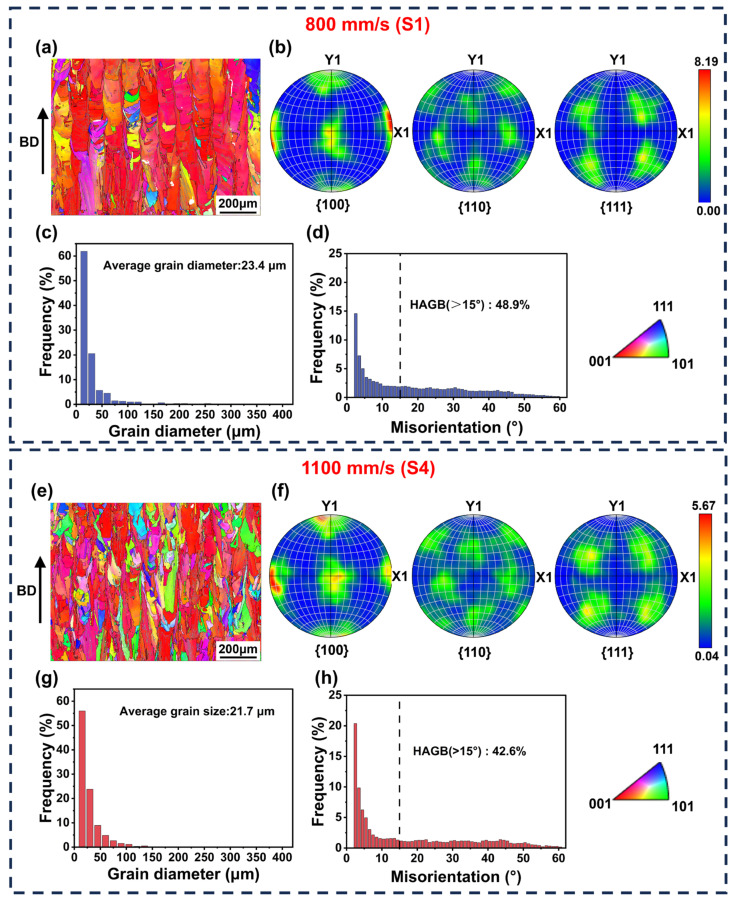
EBSD analysis along the XZ plane of the LPBF-processed IN738LC under different scan speeds: (**a**,**e**) IPF maps, (**b**,**f**) pole figure maps, (**c**,**g**) grain diameter distributions, and (**d**,**h**) grain boundary misorientation angle; (**a**–**d**) S1; (**e**–**h**) S4.

Figure 11 presents the EBSD analysis results along the BD for samples at different scan speeds. As shown in Figure 11a, S1 primarily consists of coarse columnar grains epitaxially grown along the BD, exhibiting strong <001> preferential growth characteristics. It also contains minor <101>- and <111>-oriented columnar grains, along with equiaxed grains. The corresponding pole map indicates a <001> texture strength of 8.19 (Figure 11b), suggesting highly consistent heat flow direction during solidification [39]. This strong crystallographic texture promotes the competitive growth of columnar grains. The grain boundaries within this coarse columnar structure facilitate stress concentration, creating favorable conditions for crack propagation [40]. In contrast, the S4 exhibits a typical bimodal grain morphology with coexisting columnar and equiaxed grains (Figure 11e). Compared to S1, the proportion of <001>-oriented columnar grains significantly decreased, while the number of <101>- and <111>-oriented grains increased. Grain orientation tendency toward randomization resulted in a weakened <001> texture strength of 5.67 (Figure 11f). Grain size statistics indicate that the average grain diameter of S4 decreased from 23.4 μm to 21.7 μm (Figure 11c,g). This change is attributed to altered thermal conditions in the melt pool. A higher cooling rate and a flatter melt pool morphology suppressed the epitaxial growth of columnar grains, promoting grain refinement [9,41]. Statistical analysis of grain boundary misorientation revealed that the proportion of HAGBs in S4 decreased from 48.9% to 42.6% (Figure 11d,h).

**Figure 12:**
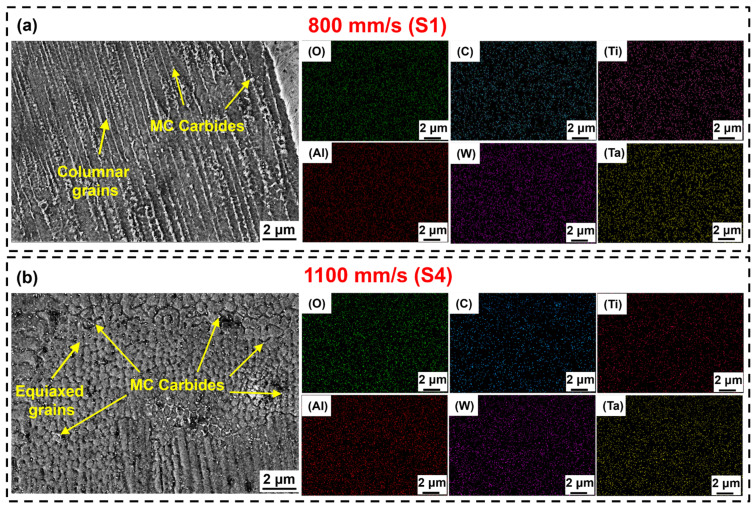
High-magnification SEM images and corresponding EDS mapping analysis of LPBF-processed IN738LC alloy at various scan speeds: (**a**) S1; (**b**) S4.

High-magnification SEM results and corresponding EDS mapping analysis are shown in Figure 12. The precipitate phases in the S1 are primarily distributed along the grain boundaries of coarse columnar grains, exhibiting sparse distribution with a broad particle size range, some exceeding 200 nm (Figure 12a). EDS analysis indicates that these particles are enriched in elements including C, Ti, Al, W, and Ta, characteristics of typical MC carbides. In contrast, fine MC carbide particles in the S4 are uniformly dispersed at the grain boundaries of equiaxed grains, with particle sizes ranging from approximately 50 to 100 nm (Figure 12b). This dispersed distribution of carbide particles contributes to improved mechanical properties of the IN738LC alloy.

### 3.3. Mechanical Properties and Fractography Behavior

**Figure 13:**
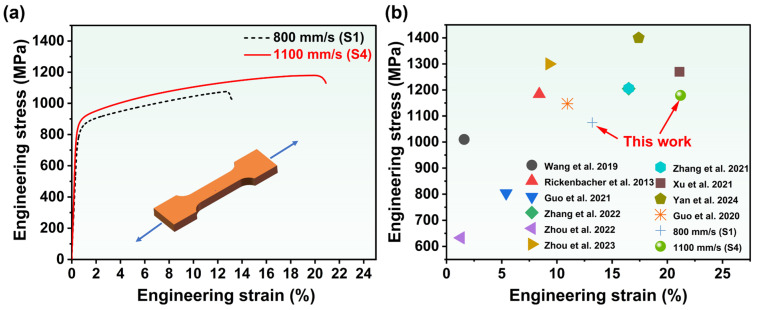
(**a**) Room-temperature engineering stress–strain curves of LPBF-processed IN738LC alloy; (**b**) comparison of UTSs and ELs from previous studies [7,10,16,18,19,23,36,38,42,43] in IN738LC alloy.

Tensile and hardness tests were conducted to research the impact of scan speed upon mechanical properties. Figure 13a displays the engineering stress–strain curves of LPBF- processed IN738LC samples at room-temperature, with corresponding yield strength (YS), ultimate tensile strength (UTS), and elongation (EL) summarized in Table 4. The study revealed that S1 exhibited lower mechanical properties, with UTS, YS, and EL values of 1075 MPa, 820 MPa, and 13.2%, respectively. In contrast, S4 demonstrated excellent strength and ductility, with UTS, YS, and EL increasing to 1179 MPa, 871 MPa, and 21.2%, respectively. Compared with previous studies, S4 achieved an outstanding balance between tensile strength and ductility (Figure 13b). Additionally, the alloy hardness increased from 365 HV_1.0_ (S1) to 387 HV_1.0_ (S4).

**Figure 14:**
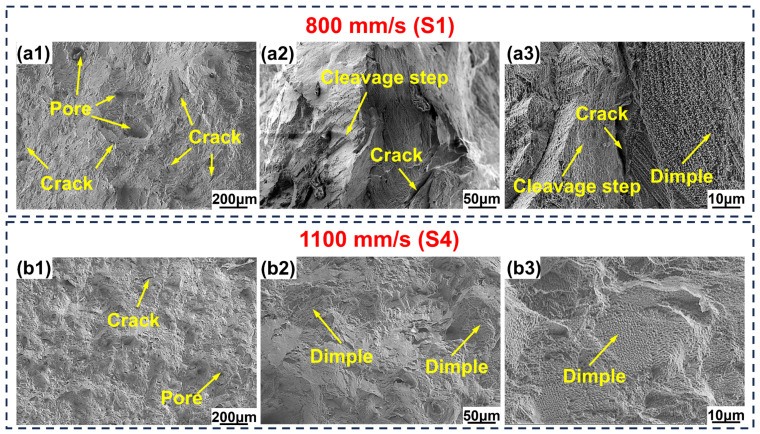
Typical tensile fracture morphology of LPBF-processed IN738LC alloy: (**a1**–**a3**) S1; (**b1**–**b3**) S4.

Figure 14 shows the tensile fracture morphology of LPBF-processed IN738LC alloy. The S1 fracture surface exhibited numerous pores and crack defects (indicated by yellow arrows in Figure 14(a1)). Figure 14(a2,a3) also revealed that the fracture surface predominantly featured cleavage steps with only a few ductile dimples, indicating brittle fracture [44]. In contrast, the S4 fracture surface exhibited a significant reduction in large pores and elongated cracks, with only minor pores and cracks remaining (yellow arrows in Figure 14(b1)). The fracture surface was characterized by numerous ductile dimples (yellow arrows in Figure 14(b2,b3)), which are typical features of ductile fracture [45].

## 4. Discussion

### 4.1. Influence of Scan Speed on Thermal History

**Figure 15:**
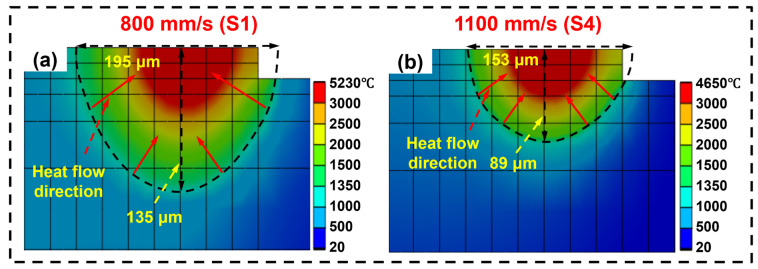
Simulation results of melt pool morphology of LPBF-processed IN738LC alloy with different scanning speeds: (**a**) S1; (**b**) S4.

Figure 15 illustrates the simulation results of the melt pool morphology of LPBF-processed IN738LC alloy with different scan speeds. As shown in Figure 15a, the lower scan speed (S1) resulted in longer laser–powder interaction time and higher melt pool temperature, forming a deeper melt pool with a simulated depth and width of 135 μm and 195 μm, respectively. Conversely, at higher scan speeds (S4), the shorter laser–powder interaction time resulted in a shallower melt pool, with a simulated depth and width of 89 μm and 153 μm, respectively (Figure 15b). The simulated melt pool size exhibits minimal deviation from experimental observations, validating the reliability of the model. Additionally, the temperature distribution within the melt pool indicated that the temperature gradient (G) was approximately perpendicular to the boundary of the melt pool and directed toward its center [46]. This indicates that the scan speed alters the thermal history of the melt pool by modifying its morphology.

**Figure 16:**
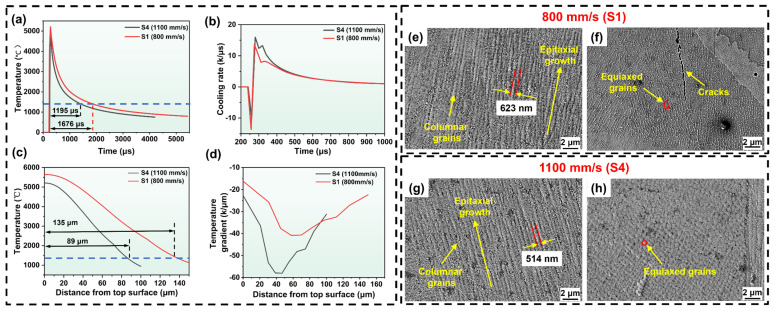
(**a**,**b**) Thermal history of the center of the third track; (**c**,**d**) temperature variation along the depth direction at the melt pool center. SEM images showing the microstructure of LPBF-processed IN738LC alloy at different scan speeds: (**e**,**f**) S1; (**g**,**h**) S4.

To investigate the impact of scan speed upon the thermal behavior, the thermal history at the center of the third track was extracted for samples at different scan speeds, as shown in Figure 16a,b. Compared to S1, the maximum temperature of the S4 melt pool decreased from 5214 °C to 4673 °C, and the lifetime of the melt pool shortened from 1676 μs to 1195 μs. Notably, node cooling rate calculations indicated that the maximum cooling rate for S4 increased from 13.68 K/μs to 15.96 K/μs compared to S1. This acceleration stemmed from the high scan speed causing a reduced melt pool size and a narrowed heat-affected zone. Consequently, heat was rapidly absorbed by the surrounding powder and substrate after laser removal, yielding a faster cooling rate [47,48]. Figure 16c displays the temperature distribution along the depth of the melt pool center, revealing a reduction in melt pool depth from 135 μm to 89 μm. Correspondingly, the G along the depth, shown in Figure 16d, exhibited a trend similar to the cooling rate changes. Compared to S1, the maximum G for S4 increased from 40.7 K/μm to 58.0 K/μm. In summary, increasing the laser scan speed reduced the maximum temperature of the melt pool, shortened its lifetime, and diminished its size, while simultaneously enhancing the cooling rate and G. Figure 16e–h compares typical solidification microstructures of LPBF-processed IN738LC alloy at different scan speeds. Compared to S1, S4 exhibited finer dendrite arm spacing and smaller cellular grain size. This was primarily attributed to the higher cooling rate induced by the increased scan speed: on the one hand, it enhanced nucleation rate during solidification; on the other hand, it shortened grain growth time [49,50].

### 4.2. Effect of Scan Speed on Cracking Behavior

Solidification cracking is considered the primary cracking mechanism in LPBF-processed Ni-based superalloys [14,20,35]. Predominantly existing at grain boundaries, their formation is primarily related to the presence of liquid films at grain boundaries and thermal stress [22,51]. The extremely rapid cooling rate in the LPBF process causes element segregation at grain boundaries, lowering the solidus temperature of the molten metal and forming a liquid film enriched with solute elements [8,15,20]. During solidification, the molten metal fills the gaps formed between dendrites; if these gaps are not completely filled, they become initiation locations for cracking. Furthermore, the dendritic coalescence theory proposed by Martin et al. [40] explains the stability of the liquid film. They indicate that when the dendrite misorientation angle is large (θ > 15°), grain boundaries exhibit repulsive characteristics, which stabilize the liquid film at low temperatures. Under thermal stress, the liquid film is sheared, resulting in crack formation. Conversely, when the dendritic misorientation angle is small (θ < 15°), grain boundaries exhibit attractive characteristics, rendering the liquid film unstable. The solid–liquid interface is displaced by grain boundaries, making crack initiation difficult.

**Figure 17:**
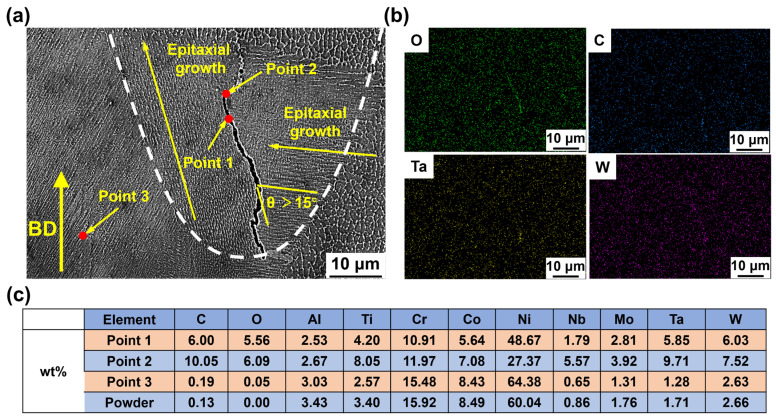
SEM images and EDS analysis of cracks in LPBF-processed IN738LC alloy: (**a**) Typical crack SEM image; (**b**) EDS plane scan near the crack; (**c**) EDS elemental analysis results at points in image (**a**).

Figure 17 illustrates the typical crack morphology and locations in LPBF-processed IN738LC alloy, indicating that solidification cracking is the primary cracking mechanism. As shown in Figure 17a, cracks initiate from the melt pool bottom and propagate along HAGBs [15]. EDS mapping analysis near the cracks confirmed the presence of O, C, Ta, and W element segregation at the crack location (Figure 17b). The EDS point analysis results of the white particles within the crack, shown in Figure 17c, confirm that these elements participate in the formation of carbide- and oxide-type brittle precipitates. Under rapid solidification conditions, the coarsening of these brittle precipitates readily induces localized stress concentration, aggravating the cracking risk [52,53]. Furthermore, carbides and oxides restrict liquid film flow, causing a localized liquid supply deficiency that further accelerates crack initiation [54].

**Figure 18:**
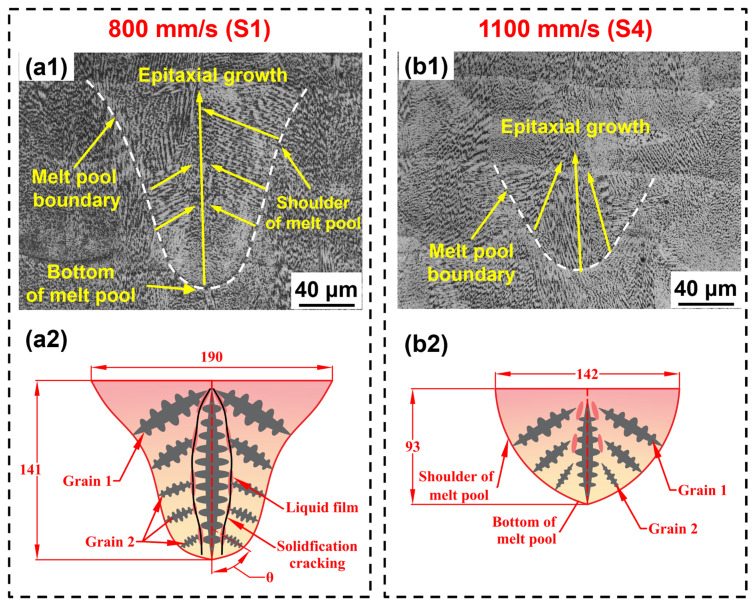
Dendrite growth within the melt pool of LPBF-processed IN738LC alloy with different scan speeds: (**a1**,**a2**) S1; (**b1**,**b2**) S4.

Dendritic growth within the melt pool is shown in Figure 18. Dendrites within the melt pool were nearly parallel to the G [55] and tend to grow outward perpendicular to the melt pool boundary from the pool bottom and shoulders (Figure 18(a1,b1)). This indicates that the scan speed controls the epitaxial growth of dendrites by altering the melt pool morphology and thermal history [46]. Schematics of dendrite growth within the melt pool, scaled to actual dimensions, are shown in Figure 18(a2,b2). The melt pool primarily contains Grain 1, which grows outward from the melt pool shoulder, and Grain 2, which grows outward from the melt pool bottom. The deep melt pool (D/W = 0.74) formed by S1 exhibited higher curvature at the shoulder, causing the outward-growing Grains 1 and 2 to meet and form a larger dendrite misorientation angle (θ). This promoted the formation of a liquid film enriched with solute elements. During the final solidification phase, thermal stress shears this liquid film, leading to crack initiation [16]. In contrast, the lower shoulder curvature of the S4 melt pool (D/W = 0.64) reduced the dendrite misorientation angle (θ), hindering the stable existence of the liquid film and suppressing crack formation. This finding was consistent with previous studies indicating that shallow melt pools help reduce crack density [22,35]. In summary, the scan speed influences the cracking behavior of IN738LC alloy by controlling the epitaxial growth of dendrites.

**Figure 19:**
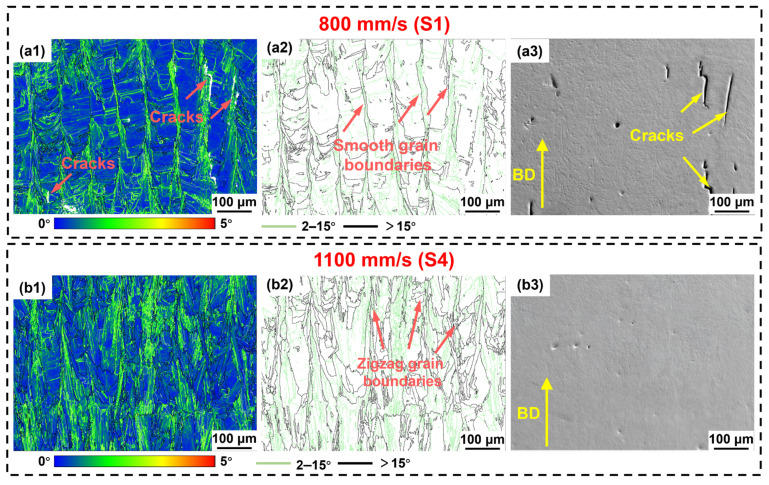
(**a1**,**b1**) Typical distributions of KAM, (**a2**,**b2**) distributions of grain boundary and (**a3**,**b3**) crack distribution of LPBF-processed IN738LC alloy at various scan speeds: (**a1**–**a3**) S1; (**b1**–**b3**) S4.

The kernel average misorientation (KAM) map serves as a measure of local strain at the microscopic scale, where bright regions indicate higher local strain and residual stress concentrations [56]. Figure 19 displays the KAM distributions for S1 and S4. As shown in Figure 19(a1,a2), high-strain regions in the S1 were primarily concentrated at the grain boundaries of coarse columnar grains. During solidification, the highly concentrated thermal stresses along these columnar grain boundaries promoted crack initiation [57]. Furthermore, the coarse columnar grain morphology in S1 reduced the number of grain boundaries, hindering effective strain dissipation. This exacerbated local stress concentration and promoted crack propagation along grain boundaries (Figure 19(a3)) [22]. Notably, although S4 exhibited higher residual stresses due to greater G (Figure 19(b1)), the refined grain morphology formed during rapid cooling dispersed stress concentration by increasing the amount of grain boundaries. Simultaneously, zigzag grain boundaries enhanced crack resistance, while the bonding between adjacent grains delayed crack propagation (Figure 19(b2,b3)). The more uniform KAM distribution in S4 indicated a more uniform strain distribution, which prevented premature failure in localized high-strain regions and significantly improved elongation. Furthermore, the scan speed may influence the crack sensitivity of the IN738LC alloy by regulating the precipitation behavior of MC carbides. As shown in Figure 12, S1 exhibits larger carbide sizes, which are prone to cause local stress concentrations and may exacerbate the risk of cracking (as shown in Figure 17). In contrast, the dispersed carbides of S4 contribute to reduced crack sensitivity. In summary, although a higher scan speed may result in greater residual stress, the significant grain refinement and the dispersed precipitation of MC carbides ultimately reduce the crack sensitivity of the IN738LC alloy.

### 4.3. Effect of Scan Speed on Mechanical Properties

Room-temperature tensile test results demonstrated that S4 (1100 mm/s) achieves a superior balance between tensile strength and ductility compared to S1 (800 mm/s). This improvement is primarily attributed to grain refinement, precipitation strengthening from carbide dispersion, dislocation strengthening, and increased EL due to crack elimination.

Both EBSD analysis and SEM results indicated reduced grain size in S4 compared to S1 (Figure 11 and Figure 16). According to the classical Hall–Petch relationship [58], the YS of the material increases with decreasing grain size. Grain refinement increases the number of grain boundaries, where dislocation motion is restricted. Consequently, higher stresses are required to activate dislocation sources within adjacent grains, thereby enhancing YS [8,19,59]. Similarly, the increase in hardness of the S4 sample (Table 4) can also be attributed to the same grain refinement effect: finer grains impede dislocation motion under indentation load, leading to enhanced local resistance to plastic deformation.

In addition to grain refinement strengthening, although S4 possesses a higher cooling rate (15.96 K/μs), which theoretically may suppress γ′ phase precipitation, the nonequilibrium microstructure formed during rapid solidification instead promoted the dispersion precipitation of MC carbides (Figure 12). During tensile deformation, these nanoscale carbide particles effectively strengthened the matrix by pinning grain boundaries and impeding dislocation motion, yielding a significant precipitation strengthening effect [8,60]. Furthermore, the dispersed carbides in S4 hinder dislocation motion during indentation, thereby increasing the hardness value.

The S4 exhibited not only enhanced strength but also a markedly improved EL (Figure 13). This improvement was mainly attributed to a notable decrease in cracking (Figure 8). As early fracture sources, cracks initiate and propagate during tensile loading, causing material failure before it reaches its inherent plasticity [61]. The abundant intergranular cracks in the S1 (800 mm/s) induced severe stress concentrations, accelerating the fracture process. Second, high residual stresses at the coarse columnar grain boundaries in the S1 (800 mm/s) further exacerbated crack propagation [19]. In contrast, crack elimination in the S4 substantially enhanced material plasticity. Furthermore, the refined grains and meandering grain boundaries in S4 effectively impeded crack propagation. KAM analysis (Figure 19) also confirmed more uniform strain distribution in S4, preventing premature failure in localized high-strain regions and significantly improving EL.

## 5. Conclusions

This study comprehensively investigated the impact of scan speed on crack elimination, microstructural evolution, and the mechanical properties of LPBF-processed IN738LC alloy through a combination of experimental and numerical simulations. The main conclusions are as follows:

(1) LPBF-processed IN738LC alloy demonstrated favorable printability within a broad process window (*VED* = 62.5–75.0 J/mm^3^). Under optimized parameters (*P* = 225 W, *v* = 1100 mm/s), near-defect-free forming was achieved, with relative density reaching 99.6%, crack density decreasing to 0.025 mm/mm^2^, and an average crack length of 33 μm.
(2) The morphology and thermal history of the melt pool were significantly altered by the scan speed. As the scan speed increased from 800 mm/s to 1100 mm/s, the melt pool morphology changed from “wine cup-shaped” (D/W = 0.74) to “semicircle-shaped” (D/W = 0.65). The cooling rate of the melt pool increased from 13.68 K/μs to 15.96 K/μs, and the G rose from 40.7 K/μm to 58.0 K/μm.
(3) Microstructural analysis revealed that the lower scan speed (S1, 800 mm/s) formed coarse columnar grains, with HAGBs accounting for 48.9%. The higher scan speed (S4, 1100 mm/s) promoted grain refinement by increasing the cooling rate, decreasing the average grain size from 23.4 μm to 21.7 μm and decreasing the HAGB proportion to 42.6%.
(4) Solidification cracking was identified as the primary mechanism. Deep melt pool promoted the formation of HAGBs, which favored the stable existence of liquid films and induced crack initiation under thermal stress. Shallow melt pools reduced the proportion of HAGBs, destabilized the liquid film, and suppressed crack initiation. Meanwhile, significant stress concentration occurred at coarse columnar grain boundaries, whereas the zigzag grain boundaries formed by refined grains effectively disperse stress, reducing cracking risk.
(5) Optimizing the scan speed achieved synergistic improvements in strength and ductility. Compared to S1 (800 mm/s), the UTS, YS, and EL of S4 (1100 mm/s) increased from 1075 MPa, 820 MPa, and 13.2% to 1179 MPa, 871 MPa, and 21.2%, respectively. Hardness increased from 365 HV_1.0_ to 387 HV_1.0_. These improvements stemmed from: grain refinement strengthening, precipitation strengthening, dislocation strengthening, and reduced crack density.

Nevertheless, this study remains subject to certain limitations. Future work will focus on the following two aspects: (1) employing three-dimensional imaging techniques, such as X-ray CT, to accurately characterize the distribution of cracks; (2) verifying the universality of this process strategy in industrial-scale multi-laser LPBF systems and other γ′-strengthened superalloys (such as CM247LC), thereby advancing the additive manufacturing applications of crack-sensitive alloys.

## Figures and Tables

**Table 1:** Composition of IN738LC powder (wt.%).

Ni	Cr	Co	W	Mo	Al	Ti	Nb	Ta	Zr	Fe	Si	C
Bal.	15.9	8.5	2.7	1.8	3.4	3.4	0.9	1.7	0.05	0.02	0.01	0.13

**Table 2:** The process parameters and corresponding *VED* of this work.

Laser Power *P* (W)	Scan Speed *v* (mm/s)				
	800	900	1000	1100	1200
175	72.9	64.8	58.3	53.0	48.6
200	83.3	74.1	66.7	60.6	55.6
225	93.8 (S1)	83.3 (S2)	75.0 (S3)	68.2 (S4)	62.5 (S5)
250	104.2	92.6	83.3	75.8	69.4
275	114.6	101.9	91.7	83.3	76.4

**Table 3:** Physical properties of IN738LC.

Properties	Value
Solidus temperature (°C)	1260 [8,14]
Liquidus temperature (°C)	1350 [8,14]
Saturation temperature (°C)	2950 [8,14]
Latent heat of fusion (J/kg)	3.0 × 10^5^ [8,14]
Latent heat of vaporization (J/kg)	6.7 × 10^5^ [8,14]
Laser absorption coefficient (%)	0.2 [8,14]
Poisson’s ratio	0.31 [13]
Thermal radiation coefficient	0.6 [31]
Convective heat transfer coefficient (W/(m^2^·K))	15 [31]

**Table 4:** A summary of the mechanical properties of the LPBF-processed IN738LC alloy.

Sample	UTS (MPa)	YS (MPa)	EL (%)	HV_1.0_
800 mm/s (S1)	1075 ± 20	820 ± 20	13.2 ± 1.7	365
1100 mm/s (S4)	1179 ± 22	871 ± 16	21.2 ± 1.8	387

## Data Availability

The original contributions presented in this study are included in the article. Further inquiries can be directed to the corresponding authors.
